# Description of two new Nematoda species of the genus *Dorylaimopsis* Ditlevsen, 1918 (Comesomatidae, Araeolaimida, Nematoda) from the Yellow Sea, China

**DOI:** 10.3897/zookeys.1277.169673

**Published:** 2026-04-16

**Authors:** Mian Huang, Xueqing Li

**Affiliations:** 1 College of Agriculture and Biology, Liaocheng University, Liaocheng 252059, China College of Agriculture and Biology, Liaocheng University Liaocheng China

**Keywords:** Biodiversity, free-living marine nematodes, meiofauna, subtidal zone, taxonomy

## Abstract

Two new species of the genus *Dorylaimopsis* Ditlevsen, 1918 from the subtidal zone of the Yellow Sea are described and illustrated. *Dorylaimopsis sinica***sp. nov**. is characterised by the lateral differentiation in the form of longitudinal rows of larger dots which consisting of three rows of dots in about the anterior 20% and posterior 20% of the total body length and two rows of dots in the rest portion of the body, by the multispiral amphidial fovea having three turns; slender, equal, arcuated spicules; cephalated proximal end with a short median cuticularised lamina; length 1.9–2.2 times the cloacal body diameter; gubernaculum with two geniculate curved apophyses and 14–17 papilliform precloacal supplements; and excretory pore posterior to nerve ring. *Dorylaimopsis zhangi***sp. nov**. is characterised by having cephalic setae 6–7 μm long, amphidial fovea with three turns, lateral differentiation consisting of four longitudinal rows of larger dots in the pharyngeal and tail regions, three longitudinal rows of larger dots in the middle region of body, spicules slender and slightly curved, length 2.0–2.5 times the cloacal body diameter, and 18 small, pit-like precloacal supplements. An updated dichotomous key is provided for the 29 valid species of *Dorylaimopsis*.

## Introduction

Free-living marine nematodes are the most widespread and abundant meiofaunal group in marine benthic habitats (Heip et al. 1982). They play a significant role in marine environment and are widely used as pollution indicators in biological monitoring (Lambshead 2004). However, many species of nematodes are still unknown. At present, less than 600 species of free-living marine nematodes have been identified in the Chinese sea area, which is estimated to be less than half of the total species (Sun et al. 2024).

The Yellow Sea is located on the edge of the western Pacific Ocean between China and the Korean Peninsula. It is a semi-enclosed inland shallow sea basin. Biodiversity surveys and taxonomical studies on nematodes in the Yellow Sea have been carried out in recent years (Gu et al. 2024; Sun et al. 2025a, 2025b). About 400 species of nematodes have been identified, of which 119 species were new to science (Sun et al. 2024). These recently described species account for 30% of the total known species. However, the total number of nematodes in this sea area is unknown, and new species are routinely found. It is therefore important to continue investigating the taxonomy of nematodes in the region.

*Dorylaimopsis* Ditlevsen, 1918 species are typical inhabitants of muddy intertidal and subtidal sediments and are often the dominant members of the nematode fauna in such habitats (Muthumbi et al. 1997; Guo et al. 2018). The genus has been reviewed successively by Jensen (1979), Zhang (1992), Leduc (2012), Guo et al. (2018), Huang et al. (2018), Fu et al. (2019), and Xiao and Guo (2023). According to Fu et al. (2019), *D. metatypica* Chitwood, 1936 should be transferred to the genus *Hopperia* because it is characterised by lateral differentiation consisting of larger, irregularly distributed coarse dots, as in *Hopperia*, and not longitudinal rows of coarse dots, as in *Dorylaimopsis*. We also agree with this opinion and consider that this species does not belong to the genus *Dorylaimopsis*. To date, 29 valid species have been recorded worldwide (Fu et al. 2019; Xiao and Guo 2023; Nemys Eds 2025). Seven species of the genus have been found in the sea of China: *D. boucheri* Fu, Leduc, Rao & Cai, 2019, *D. heteroapophysis* Huang, Sun & Huang, 2018, *D. jinmendaoica* Xiao & Guo, 2023, *D. longispicula* Fu, Leduc, Rao & Cai, 2019, *D. papilla* Guo, Chang & Yang, 2018, *D. rabalaisi* Zhang, 1992, and *D. turneri* Zhang, 1992). Here, we describe two new species of *Dorylaimopsis* that were discovered in the subtidal zone of the Yellow Sea.

## Materials and methods

Seafloor sediments were collected using a 0.1 m^2^ improved Gray-O’Hara box corer from a grid of sampling stations between 32°00'N and 38°50'N, and between 120°E and 124°E during the Open Research Cruise offshore China in 2018. Meiofauna samples were obtained from the top sediment layer (0–8 cm deep) using a 2.9 cm diameter sawn-off syringe. The samples were fixed with an equal amount of 10% formalin solution. In laboratory, samples were stained with 0.1% Rose Bengal for 24 h. The stained samples were poured through two sieves (500 and 42 µm mesh sizes) and washed with tap water to remove silt and separate macrofauna from meiofauna. The retained material in the 42 µm mesh was centrifuged in Ludox-TM (50% colloidal silica, suspension in water; product of Sigma Aldrich Co., USA) with a specific gravity of 1.15 g/ml (de Jonge and Bouwman 1977) to separate meiofauna from the heavier sediment particles. The supernatant obtained by centrifugation for three times was poured into the 42 µm mesh to filter Ludox-TM. The sample in the 42 µm mesh was washed into a Petri dish with distilled water and meiofauna was sorted out under a stereoscopic microscope.

Nematodes were transferred into a cavity block containing a solution of 5% glycerol, 5% pure ethanol, and 90% distilled water in volume (McIntyre and Warwick 1984). After ethanol was slowly evaporated, the specimens were mounted in glycerine on permanent slides. The descriptions were made using a differential interference contrast microscope (Leica DM 2500). Photographs were taken with a Leica DMC 5400 digital camera. Line drawings were made with the aid of a camera lucida. All measurements were taken using Leica software of LAS X v. 3.3.3, and all curved structures were measured along the curved median line. All measurements are in μm. Type specimens were deposited in the Marine Biological Museum of the Chinese Academy of Sciences, Qingdao.

## Results and discussion

### Order Araeolaimida De Coninck & Schuurmans Stekhoven, 1933

**Family Comesomatidae Filipjev, 1918**

#### Genus Dorylaimopsis

Taxon classificationAnimaliaAraeolaimidaComesomatidae

Ditlevsen, 1918

2BDC7E4D-529F-54F4-BCAC-46E1FB047EBF

##### Diagnosis.

(from Platt and Warwick 1988; Leduc 2012) Cuticle with transverse rows of punctations, lateral differentiation with longitudinal rows of coarse dots; buccal cavity cylindrical with three or six teeth; six outer labial sensilla and four cephalic setae in separate circles; spicules slender, usually elongated, varying from sinusoid to slightly bent or curved; gubernaculum with caudal or dorso-caudal apophyses; precloacal supplements usually present; tail conico-cylindrical with terminal setae.

###### List of valid species

*D. angelae* Inglis, 1968

*D. boucheri* Fu, Leduc, Rao & Cai, 2019

*D. brevispiculata* Gagarin, 2013

*D. communis* (Gagarin & Nguyen, 2006) Fu, Leduc, Rao & Cai, 2019

*D. coomansi* Muthumbi, Soetaert & Vincx, 1997

*D. gerardi* Muthumbi, Soetaert & Vincx, 1997

*D. halongensis* Nguyen, Nguyen, Smol & Vanreusel, 2008

*D. heteroapophysis* Huang, Sun & Huang, 2018

*D. intermedia* Gagarin, 2013

*D. janetae* Inglis, 1963

*D. jinmendaoica* Xiao & Guo, 2023

*D. jinyuei* Fu, Leduc, Rao & Cai, 2019

*D. longispicula* Fu, Leduc, Rao & Cai, 2019

*D. lutosa* Gagarin, 2017

*D. magellanense* Chen & Vincx, 1998

*D. mediterranea* Grimaldi-De Zio, 1968

*D. nini* Inglis, 1961

*D. nodderi* Leduc, 2012

*D. papilla* Guo, Chang & Yang, 2018

*D. peculiaris* Platonova, 1971

*D. pellucidum* Cobb, 1920

*D. perfecta* Cobb, 1920

*D. poriferum* Cobb, 1920

*D. punctata* Ditlevsen, 1918

*D. rabalaisi* Zhang, 1992

*D. timmi* Jensen, 1979

*D. tumida* Gagarin & Nguyen Vu Thanh, 2006

*D. turneri* Zhang, 1992

*D. variabilis* Muthumbi, Soetaert & Vincx, 1997

#### Dorylaimopsis sinica sp. nov.

Taxon classificationAnimaliaAraeolaimidaComesomatidae

8CD04AB8-F550-54D9-9A46-44B16AC787D5

https://zoobank.org/F4E1CF5A-2407-4BF2-8D90-9BE362F1ADFB

Figs 1, 2, Table 1

##### Holotype and paratype material.

Five males and three females were measured. Holotype male and paratype male 2, female 1 and 2 are on the slide YS3600-4-2-1, paratype male 3 and female 3 are on the slide YS3600-4-2-3, paratype male 4 and male 5 on the slide YS3600-4-5-1.

**Figure 1.**
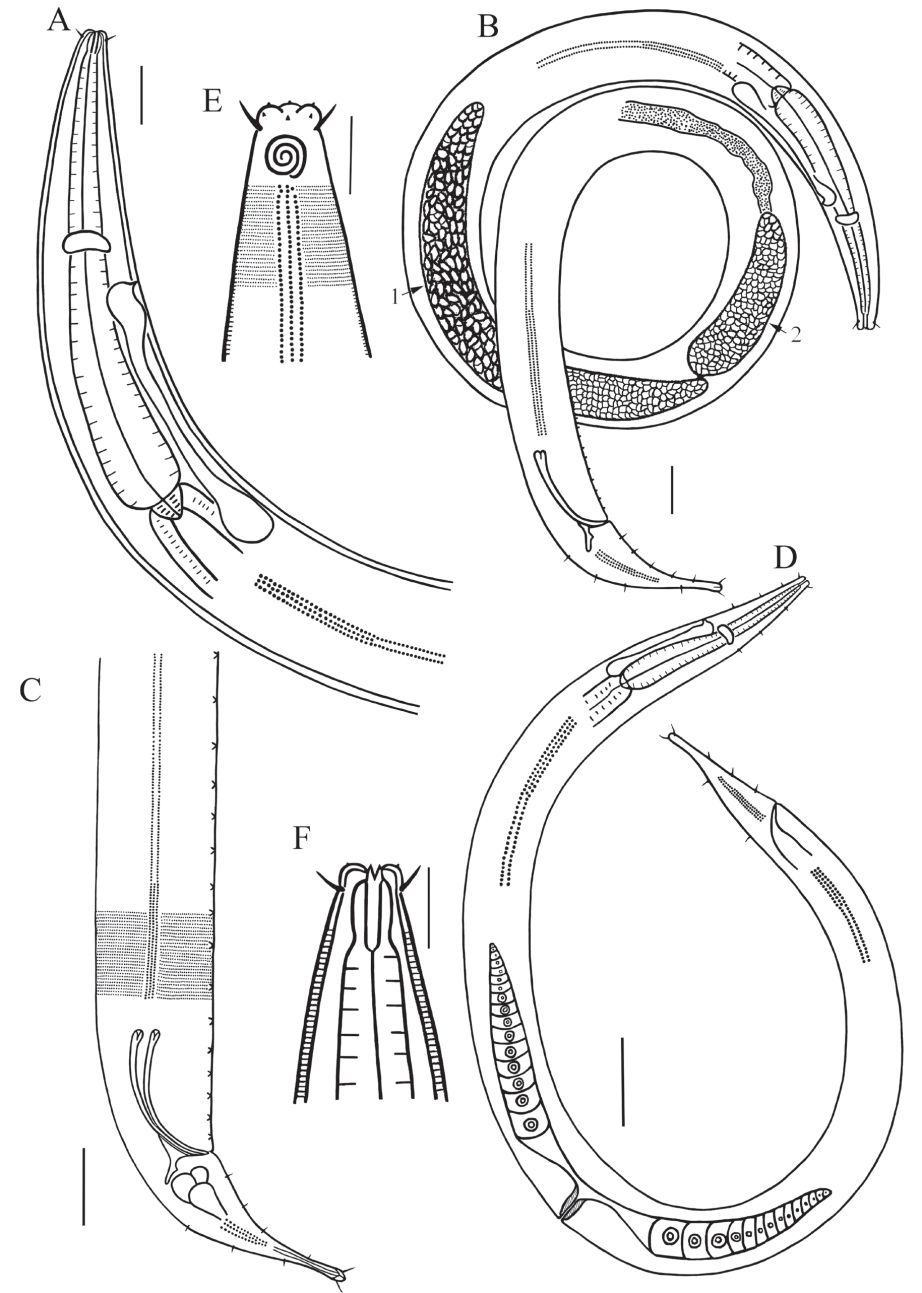
Drawings of *Dorylaimopsis sinica* sp. nov. **A**. Pharyngeal region of holotype male; **B**. Entire view of holotype, arrow 1 indicates anterior testis, arrow 2 indicates posterior testis; **C**. Posterior end of holotype male, showing spicules, gubernaculum, precloacal supplements and lateral differentiation; **D**. Entire view of female, showing reproduction system; **E**. Surface view of anterior end of male, showing amphidial fovea, lateral differentiation and anterior sensilla; **F**. Anterior end of male, showing cephalic setae and buccal cavity. Scale bars: 50 µm (**A, B, C**); 100 µm (**D**); 20 µm (**E, F**).

**Figure 2.**
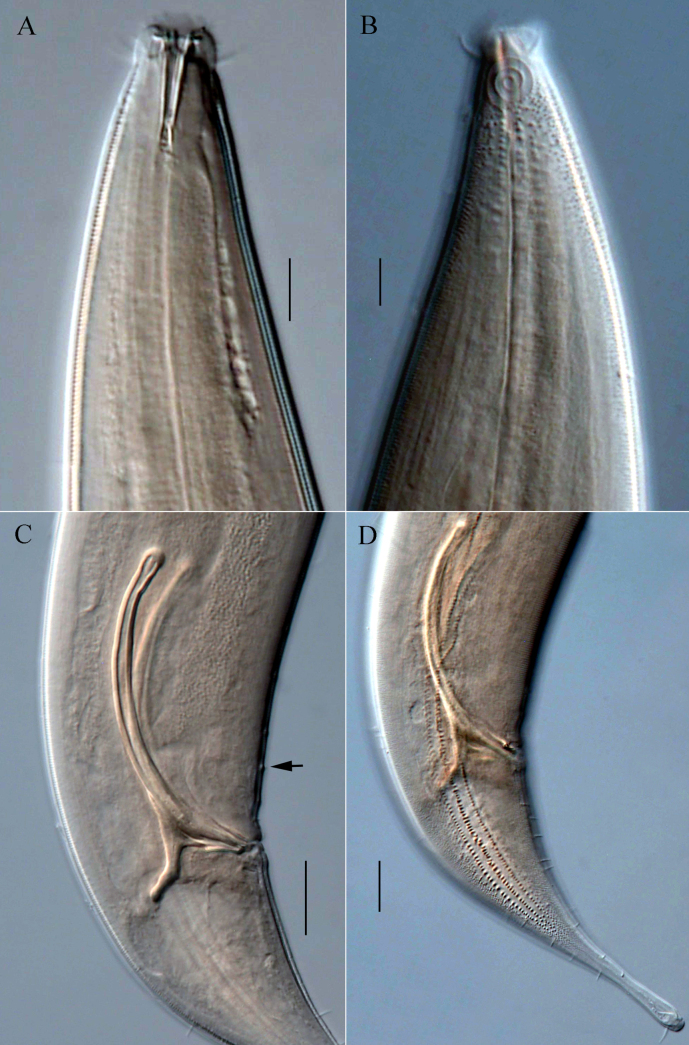
Microphotographs of *Dorylaimopsis sinica* sp. nov. **A**. Anterior end of holotype, showing buccal cavity with teeth; **B**. Surface view of anterior end of male 2, showing cephalic setae and amphidial fovea; **C**. Cloacal region of holotype, showing spicules, gubernaculum and precloacal supplements (arrow); **D**. Posterior end of holotype, showing lateral differentiation, caudal setae and terminal seta. Scale bars: 10 µm (**A, B**); 20 µm (**C, D**).

**Table 1.** Individual measurements of *Dorylaimopsis sinica* sp. nov. and *Dorylaimopsis zhangi* sp. nov. (in µm except a, b, c and V%). a, ratio of body length to maximum body diameter; b, ratio of body length to pharynx length; c, ratio of body length to tail length; c', ratio of tail length to cloacal or anus body diameter; V%, position of vulva from anterior end expressed as a percentage of total body length; –, no data.

Characters	*Dorylaimopsis sinica* sp. nov.								*D. zhangi* sp. nov.		
	holotype	♂2	♂3	♂4	♂5	♀1	♀2	♀3	holotype	♂2	♂3
Total body length	2110	2210	2150	2192	2263	2158	2158	2090	2053	1970	1956
Maximum body diameter	71	106	68	78	97	76	88	87	59	66	77
Head diameter	13	14	14	14	15	13	15	14	14	15	15
Length of cephalic setae	5	6	5	6	7	7	6	7	6	6	7
Diameter of amphidial fovea	10	10	10	10.5	11	9	9	9	10	–	10
Body diameter at amphidial fovea	15	16	17	18	18	15	17	17	16	–	18
Excretory pore from anterior end	143	149	163	144	142	150	140	144	162	150	–
Nerve ring from anterior end	110	116	135	–	116	121	128	122	139	–	–
Body diameter at nerve ring	49	63	51	–	56	48	61	57	49	–	–
Length of pharynx	254	257	276	260	246	252	257	260	247	260	252
Body diameter at pharyngeal base	66	85	66	75	76	67	81	78	56	62	74
Spicules length along arc	104	114	116	113	111	–	–	–	118	112	109
Length of gubernacular apophyses	18	19	20	20	21	–	–	–	17	18	18
Number of precloacal supplements	16	17	–	15	14	–	–	–	18	18	18
Vulva from anterior end	–	–	–	–	–	1075	1076	992	–	–	–
Body diameter at vulva	–	–	–	–	–	76	81	84	–	–	–
V%	–	–	–	–	–	49.8	49.9	47.5	–	–	–
Body diameter at cloaca or anus	52	60	52	54	–	49	54	58	47	50	55
Tail length	154	151	163	167	161	169	164	172	156	179	150
a	29.7	20.8	31.6	28.1	23.3	28.4	24.5	24.0	34.8	29.8	25.4
b	8.4	8.6	7.9	8.4	9.2	8.6	8.4	8.0	8.3	7.6	7.8
c	13.7	14.6	13.2	13.1	14.1	12.8	13.2	12.2	13.2	11.0	13.0
c'	3.0	2.5	3.1	3.1	2.8	3.4	3.0	3.0	3.3	3.6	2.7

##### Type locality and habitat.

Holotype and paratype male and females were collected from the sea floor muddy sediment at Station 3600-4 in the Yellow Sea, 36°N, 122°E, water depth 36 m, water temperature at the sediment–water interface 9.98 °C, salinity 33.6.

##### Etymology.

The species epithet *sinica* refers to the country of origin, China.

##### Measurements.

All measurement data are given in Table 1.

##### Description.

**Males**. Body cylindrical, tapering slightly towards extremities. Cuticle marked by transverse rows of fine punctations. Lateral differentiation consists of two or three longitudinal rows of larger dots. Three longitudinal rows of larger dots beginning just posterior to amphidial fovea, extending down to anterior part of intestine and then taking the form of two longitudinal rows of dots until anterior to cloaca. Remaining posterior part of body with three longitudinal rows of dots, similar on pharyngeal region, terminating at junction of conical and cylindrical parts of tail. Anterior ~20% and posterior 20% of total body length with lateral differentiation in the form of three longitudinal rows of dots; rest of body with two longitudinal rows of dots. Lateral differentiation about 10 µm wide at mid-body, 14% of corresponding body diameter. Head set off by a contraction at level of cephalic setae. Head sensilla arranged in 6+6+4 pattern. Inner labial and outer labial sensilla papilliform. Four cephalic setae 5–6 µm long. Somatic setae scattered mainly on cervical region, 2–3 µm long. Amphidial fovea multispiral, of three turns, 10 µm wide, and occupies about 59–63% of the body diameter at its level. It is located immediately posterior to cephalic setae. Anterior conical part of buccal cavity with three triangular teeth; posterior cylindrical part with strongly sclerotised walls, 19–20 μm deep and 3–4 μm wide. Pharynx largely cylindrical but with widened base, not forming true bulb. Nerve ring anterior to middle of pharynx. Excretory system in mid-posterior portion of the pharynx, with excretory pore opening posterior to nerve ring, 143–163 µm from anterior end. Ventral gland cell oval, located at the level of cardia. Cardia conical, 11 μm long and embedded in intestine. Tail conico-cylindrical, with numerous conical setae, 4–6 μm long. Tail tip slightly enlarged, with three terminal setae, 5– 6 μm long. Caudal glands three, and distinct spinneret present.

Reproductive system diorchic, with two opposed, outstretched testes. Anterior testis longer (about 340 μm, with some larger sperm cells), located to left of intestine. Posterior testis shorter (about 170 μm, with smaller sperm), located to right of intestine. Spicules slender, equal, and arcuated, length 1.9–2.2 times the cloacal body diameter. Proximal end cephalated, with a short, median, cuticularised lamina at proximal tip; distal end tapered. Gubernaculum with two caudo-dorsal apophyses, 18–21 µm long, and apophyses geniculate, curved. Precloacal supplements 14–17, small, papilliform, extending forward from 12–290 μm anterior to cloaca; distance between adjacent supplements 10–20 μm, gradually increasing anteriorly.

**Females**. Similar to males in general characteristics, but tail slightly longer (c' = 3.4 vs 2.5–3.0). Reproductive system amphidelphic, with two opposed, outstretched ovaries. Anterior ovary to left, and posterior gonad to right, of intestine. Anterior ovary apex approximately 410 μm from vulva; posterior ovary apex 450 μm from vulva. Vulva at mid-body. Vagina short and with thick walls. Spermatheca not observed.

##### Diagnosis.

*Dorylaimopsis sinica* sp. nov. is characterised by the lateral differentiation in the form of longitudinal rows of larger dots which consist of three rows of dots in about the anterior 20% and posterior 20% of the total body length, and two rows of dots in the rest portion of body; the amphidial fovea mutispiral has three turns; the buccal cavity is cylindrical and with three teeth; spicules are slender, equal, and arcuated; the proximal end is slightly cephalated and with a short median cuticularised lamina, and the distal end is tapered; the gubernaculum bears two caudo-dorsally geniculate curved apophyses and 16 or 17 papilliform precloacal supplements; the excretory pore is posterior to the nerve ring.

##### Differential diagnosis.

The new species resembles *D. brevispiculata*, *D. heteroapophysis*, *D. nodderi*, *D. papilla*, *D. punctata*, *D. rabalaisi*, and *D. variabilis* in having the same lateral differentiation pattern of longitudinal rows of dots (3-2-3 pattern). However, it differs from *D. brevispiculata* in its longer body length (2110–2210 μm vs 1234–1758 μm), longer spicules (104–116 μm vs 59–68 μm), geniculate gubernacular apophyses (vs straight and 20–27 μm long), and different shape of the outer labial sensillae (papillae vs long setae). The new species differs from *D. heteroapophysis* in having longer spicules (104–116 μm vs 58–69 μm), pairs of gubernacular apophyses of equal length of (vs unequal in the latter species), and different precloacal supplements (16, papilliform vs 11–12, fine and tubular). The new species differs from *D. papilla*, in having longer cephalic sensilla (5–7 μm long and 38–54% head diameter vs 3.7–4.2 μm, 22%–24%); amphidial fovea with three turns (vs 2.5 turns), a gubernaculum with shorter, geniculate, curved apophyses (18–20 μm vs 37–40 μm, almost straight in the latter species), and a conico-cylindrical tail with its posterior third cylindrical (vs tail with posterior half thin and cylindrical). *Dorylaimopsis sinica* sp. nov. differs from *D. punctata* by its shorter body length (2110–2210 μm vs 2484–3221 μm), shorter spicules without a ventral apophysis (104–116 μm vs 154–182 μm, with a ventral apophysis) and geniculate gubernacular apophyses (vs 20–27 μm, almost straight). The new species differs from *D. rabalaisi* in having longer spicules (104–116 μm vs 60–97 μm), shorter gubernacular apophyses (18–20 μm vs 24 μm), and different shape of precloacal supplements in males (papilliform supplements vs tubular supplements). The new species differs from *D. variabilis* in having differently shaped precloacal supplements in males (papilliform supplements vs tubular supplements), relatively shorter gubernacular apophyses (32–38% of cloacal body diameter vs 48–66%), different spicule structure, and different type of spermatozoa (spermatozoa almost the same in size in anterior and posterior testes vs two types of spermatozoa, a large type in anterior testis and a small one in the posterior testis in *D. variabilis*). The new species is closely similar to *D. lutosa* in having two longitudinal rows of lateral coarse dots in the middle portion of the body, but it is distinguished from the latter species by the lateral differentiation with three longitudinal rows of coarse dots in the pharynx, precloacal, and preanal regions, and on tail, while the latter species with 4–6 such longitudinal rows of coarse dots in the corresponding portion. In addition, *D. lutosa* has longer and slender tail (c' = 4.2–5.5).

#### Dorylaimopsis zhangi sp. nov.

Taxon classificationAnimaliaAraeolaimidaComesomatidae

729A77DD-B29C-5E00-8F9D-C1D441F9FDC4

https://zoobank.org/7EDF105B-F59C-418A-A278-4EED8D28F641

Figs 3, 4, Table 1

##### Holotype and paratype material.

Three males were measured. Holotype male 1 and paratype male 2 are on the slide YS3825-3-0-2, paratype male 3 is on the slide YS3825-2-0-2.

**Figure 3.**
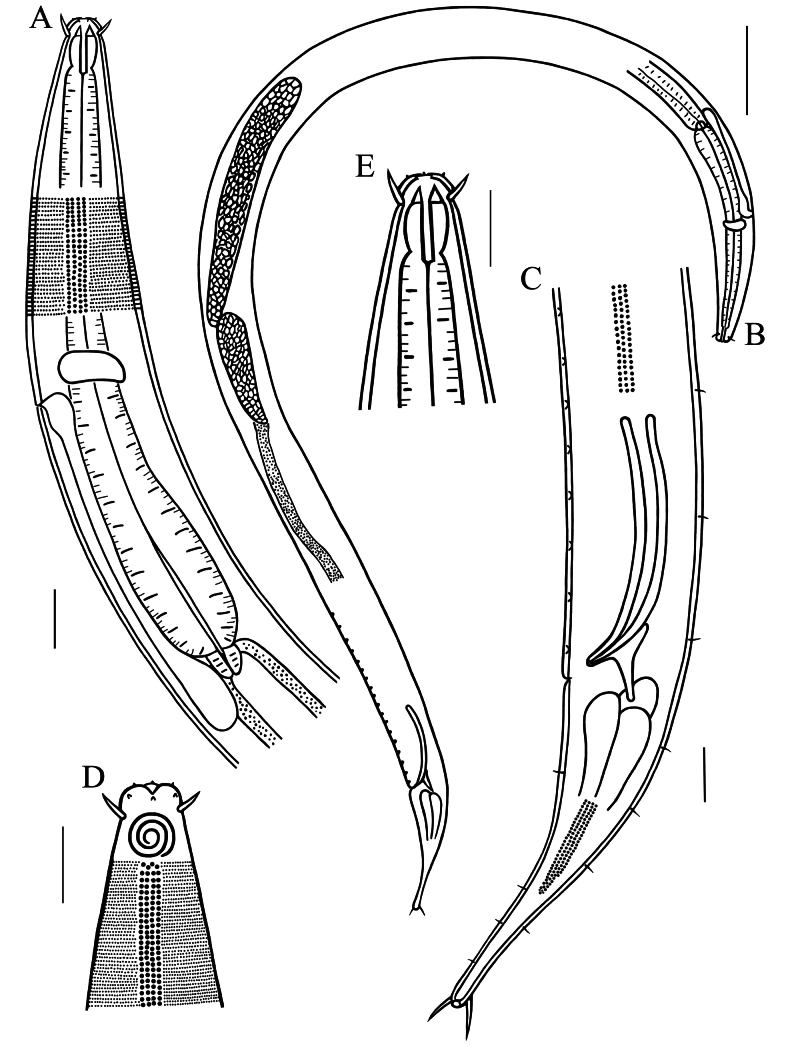
Drawings of *Dorylaimopsis zhangi* sp. nov. **A**. Pharyngeal region of male; **B**. Entire view of holotype, showing anterior and posterior testes; **C**. Posterior end of holotype, showing spicules, gubernaculum, precloacal supplements and tail; **D**. Surface view of anterior end of male, showing cephalic setae, amphidial fovea and lateral differentiation; **F**. Anterior end of male, showing cephalic setae and buccal cavity. Scale bars: 20 µm (**A, C, D, E**); 100 µm (**B**).

**Figure 4.**
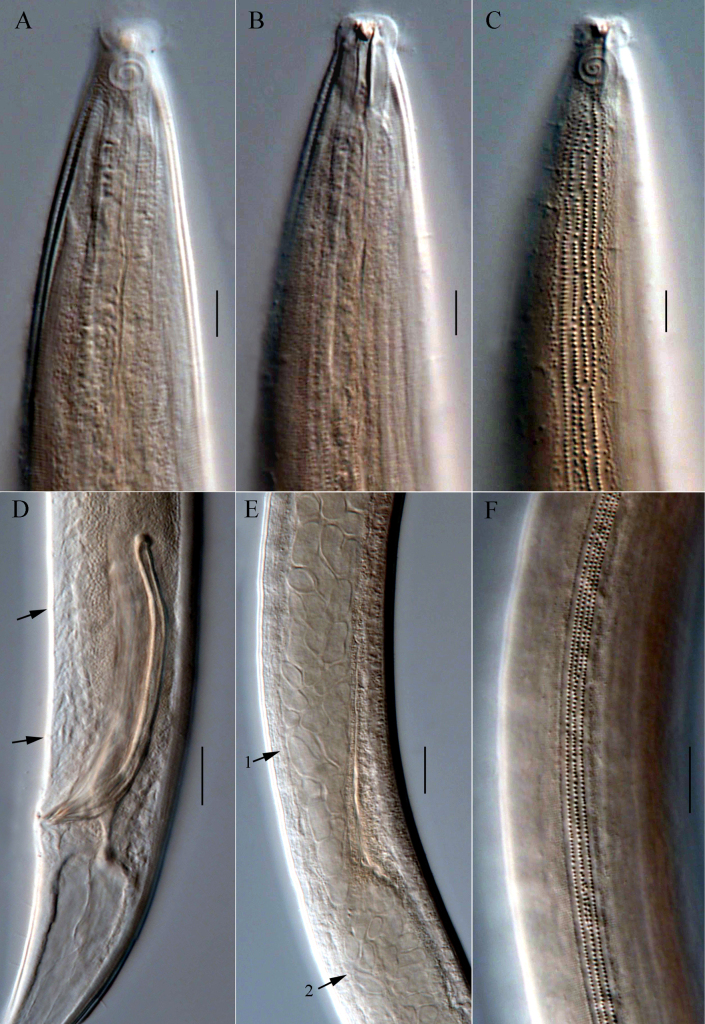
Microphotographs of *Dorylaimopsis zhangi* sp. nov. **A**. Anterior end of holotype, showing cephalic setae and amphidial fovea; **B**. Anterior end of holotype, showing buccal cavity with teeth; **C**. Anterior end of holotype, showing lateral differentiation; **D**. Cloacal region of holotype, showing spicules, gubernaculum and precloacal supplements (arrows); **E**. Middle region of holotype, showing anterior testis (arrow 1) and posterior testis (arrow 2); **F**. Surface view of the middle of holotype, showing lateral differentiation. Scale bars: 10 µm (**A, B, C**); 20 µm (**D, E, F**).

**Table 2.** Individual measurements of the differences between *D. sinica* sp. nov. and closest congeneric species (in µm; hd, head diameter; —, no data).

Characters	*D. sinica*	*D. brevispiculata*	*D. papilla*	*D. punctata*
Total body length	2110–2210	1234–1758	2008–2392	2484–3221
Outer labial sensillae	papillae	setae	papillae	papillae
Cephalic sensilla (% hd)	38–54	8–9.5	22–24	9.5–13.4
Amphidial fovea	3 turns	3 turns	2.5 turns	3.5 turns
Spicules length	104–116	59–68	101–107	154–182
Gubernacular apophyses	geniculate equal in length	straight equal in length	straight equal in length	straight equal in length
Precloacal supplements	14–17 papilla	–	16–18 papillate	18–22 papilla
Tail	conico-cylindrical	conico-cylindrical	filiform	conico-cylindrical

**Table 3.** Individual measurements of the differences between *D. zhangi* sp. nov. and closest congeneric species (in µm; hd, head diameter; cbd, corresponding body diameter).

Characters	*D. zhangi*	*D. papilla*	*D. longispicula*	*D. janetae*
Male body length	2110–2210	2019–2392	2061–2252	4800–5600
Cephalic sensilla (%hd)	43–47	22–24	56–64	8
Amphidial fovea	3 turns	2.5 turns	3 turns	2.5 turns
Precloacal supplements	pit-like	papilliform	tubular	–
Pattern of lateral differentiation	4-3-4	(4 or 5)-2-4	4-2-4	4-3
Length of spicules	2–2.5 cbd	1.5–1.8 cbd	179–197	220
Length of gubernacular apophyses	17–18	37–40	29–41	75–78

##### Type locality and habitat.

Holotype and paratypes were collected from the sea floor muddy sediment at Station 3825-2 in the Yellow Sea, 38°25'N, 122°40'E, water depth 55 m.

##### Etymology.

The specific epithet *zhangi* is in honour of Professor Zhinan Zhang, a Chinese nematologist.

##### Measurements.

All measurement data are given in Table 1.

##### Description.

**Males**. Body cylindrical, anteriorly with blunt end, and with a conico-cylindrical tail. Punctuation begins behind anterior border of amphidial fovea. Cuticle marked by transverse rows of fine punctuation, with lateral differentiation in the form of longitudinal rows of enlarged dots, running from posterior to amphidial fovea in distinct four lines, three lines at mid-body, then increasing to four lines in spicule and tail regions. Lateral differentiation about 8 μm wide in pharyngeal region, 5 μm wide in middle of body, about 8.5% of corresponding body diameter. On dorsal and ventral sides, punctuations smaller and arranged in transverse rows. Anterior sensilla in three circles; inner and outer labial sensilla papilliform; cephalic sensilla setiform, 6–7 μm long, and 43–47% of head diameter. Amphidial fovea spiral, with three turns, and located immediately posterior of cephalic sensilla. Cephalic sensilla 9–10 μm in diameter (56%–63% of corresponding body diameter). Anterior conical part of buccal cavity with three triangular teeth; posterior cylindrical part with strongly sclerotised walls, 18–20 μm deep, and 3 μm wide. Nerve ring at about 56% of pharynx length. Excretory system in mid-posterior portion of pharynx, with excretory pore opening just posterior to nerve ring, 150–123 µm from anterior end. Ventral gland cell oval, located at the level of cardia. Cardia conical, 13 µm long.

Reproduction system with two opposite and outstretched testes. Anterior testis longer, 305 μm long, with many larger sperm. Posterior testis shorter, 130 μm long, with smaller sperm. Spicules slender, slightly curved, length 2.0–2.5 times the cloacal body diameter. Proximal end not enlarged. Gubernaculum with paired equal dorso-caudal apophyses, 17–18 μm long; apophyses end rounded. Precloacal supplements about 18, small, cavity-like; distances between precloacal supplements almost as equal. Tail conico-cylindrical, length 2.7–3.6 times the cloacal body diameter; anterior half of tail conical, posterior half cylindrical. Numerous setae, 4–5 μm long, on conical part of tail; three setae at tip, 9–10 μm long. Three caudal glands present with one extending beyond gubernaculum apophyses.

**Females**. Not found.

##### Diagnosis.

*Dorylaimopsis zhangi* sp. nov. is characterised by having cephalic setae 6–7 μm long, amphidial fovea with three turns, lateral differentiation consisting of four longitudinal rows of larger dots in the pharyngeal and tail regions, three longitudinal rows of larger dots in the middle region of the body, slender, slightly curved spicules, 2.0–2.5times the cloacal body diameter in length, and about 18 small pit-like precloacal supplements.

##### Differential diagnosis.

The new species is similar to *D. papilla*, but it differs by its longer cephalic sensilla (6–7 μm long and 43%–47% head diameter vs 3.7–4.2 μm, 22%–24%); amphidial fovea with three turns (vs 2.5 turns), precloacal supplements pit-like (vs papilliform), lateral differentiation in three rows in the middle region of body (vs two rows) then increasing to four or more rows in the spicule region, spicules longer (length 2.0–2.5 times the cloacal body diameter, not enlarged proximally vs 1.5–1.8 times the cloacal body diameter long and with an enlarged proximal end), and gubernacular apophyses shorter (17–18 μm vs 37–40 μm long). The new species is close to *D. longispicula* in having lateral differentiation consisting of four longitudinal rows of larger dots in pharyngeal and tail regions, but it differs in having the lateral differentiation consisting of three rows of larger dots in the middle regions, while two rows of larger dots in the middle regions in the latter species, cephalic setae shorter (6–7 μm vs 8–11 μm long in the males), shorter spicules (109–118 μm vs 179–196 μm long), and shorter gubernacular apophyses (17–18 μm vs 29–41 μm long). In having three rows of lateral coarse dots in middle body region, the new species is closely similar to *D. janetae*, but it differs from the latter species in having a longer body and longer spicules (body length 4.8–5.6 mm and spicules length 220 μm). In addition, *D. zhangi* sp. nov differs from *D. sinica* sp. nov. in having different types of lateral differentiation (4+3+4 vs 3+2+3 in the latter species), spicules not enlarged proximally and without median lamina vs with an enlarged proximal end and a short median lamina in the latter species, gubernacular apophyses straight vs geniculate curved, and 18 small, pit-like precloacal supplements vs 14–17 papilliform precloacal supplements in *D. sinica* sp. nov.

### Updated dichotomous key to the valid species of *Dorylaimopsis* Ditlevsen, 1918 (based on Fu et al. 2019)

Abbreviations: a, ratio of body length to maximum body diameter; abd, body diameter at cloaca or anus; c', ratio of tail length to cloacal or anus body diameter.

**Table:** 

1	Lateral differentiation of cuticle varies within one species, cuticle with four longitudinal rows of coarse dots in lateral fields in anterior half or entire body, or with lateral irregularly distributed dots	***D. communis***
–	Cuticle always with longitudinal rows of dots in part or over entire body	**2**
2	Cuticle with lateral longitudinal rows of coarse dots along part of body length	**3**
–	Cuticle with longitudinal rows of coarse dots along entire length of body	**5**
3	Lateral longitudinal rows of coarse dots present posterior to pharyngeal region only	**4**
–	Lateral longitudinal rows of coarse dots present in two regions, one is from posterior edge of amphid to anterior of intestine region, the other is tail region in males	***D. jinyuei***
4	Lateral differentiation of cuticle consisting of 1 – 3 longitudinal rows of dots, gubernacular apophyses with swollen distal end	***D. coomansi***
–	Lateral differentiation of cuticle consisting of five longitudinal rows of dots, gubernacular apophyses tapering distally	***D. turneri***
5	Cuticle with two longitudinal rows of coarse dots in the middle region of body in both sexes	**6**
–	Cuticle with more than two longitudinal rows of coarse dots in the middle body at least one of the sexes	**25**
6	Spicules articulated in the middle and divided into two equal segments	***D. perfecta***
–	Spicules not articulated and not divided into two segments	**7**
7	Spicules with ventral projection, giving appearance of a joint	**8**
–	Spicules without ventral projection	**9**
8	Body shorter than 1700 μm, cuticle with three rows of coarse dots in pharyngeal and caudal regions	***D. nodderi***
–	Body longer than 1900 μm, cuticle with two rows of coarse dots in pharyngeal and caudal regions	***D. punctata***
9	Spicules hooked proximally, gubernacular apophyses with pointed end	***D. peculiaris***
–	Spicules not hooked proximally, gubernacular apophyses with rounded end except *D. papilla*	**10**
10	Spicules longer than 140 μm, strongly curved	**11**
–	Spicules shorter than 130 μm, arcuated	**13**
11	Spicules double ventral curved, W-shaped in appearance	**12**
–	Spicules straight in anterior part and irregularly curved in posterior part	***D. longispicula***
12	Spicules longer than 180 μm, lateral differentiation with 3 longitudinal rows of dots in pharyngeal and cloacal regions	***D. mediterranea***
–	Spicules shorter than 170 μm, lateral differentiation with 4 longitudinal rows of dots in pharyngeal and cloacal region	***D. intermedia***
13	Lateral differentiation with 2 longitudinal rows of coarse dots in the pharyngeal region, body shorter than 1810 μm, spicules 75–85 μm long	***D. tumida***
–	Lateral differentiation with 3–5 longitudinal rows of dots in the pharyngeal region	**14**
14	Gubernacular apophyses 12–17 μm, spicules with poorly developed capitulum, tail 2.7 abd long	***D. gerardi***
–	Gubernacular apophyses longer than 18 μm, tail longer than 2.7 abd	**15**
15	Lateral differentiation consisting of 3 longitudinal rows of dots in the pharyngeal region	**16**
–	Lateral differentiation consisting of 4–5 longitudinal rows of dots in the pharyngeal region	**21**
16	Spicules longer than 100 μm	**17**
–	Spicules shorter than 90 μm	**18**
17	Gubernaculum with geniculate curved apophyses, 18 – 20 μm long	***D. sinica* sp. nov**.
–	Gubernaculum with straight apophyses, 23 – 38 μm long	***D. variabilis* (population 1)**
18	Precloacal supplements absent	***D. brevispiculata***
–	Precloacal supplements present	**19**
19	Spicules with median cuticularised strip proximally, two gubernacular apophyses unequal	***D. heteroapophysis***
–	Spicules without median cuticularised strip proximally, two gubernacular apophyses equal	**20**
20	Spicules slender without well-developed capitulum, gubernacular apophyses 24 μm	***D. rabalaisi***
–	Spicules broad with well-developed capitulum, gubernacular apophyses 15–23 μm	***D. variabilis* (population 2)**
21	Spicules with small distal hook, cephalic setae longer than head diameter	***D. pellucida***
–	Spicules without distal hook, cephalic setae shorter than head diameter	**22**
22	Spicules 82 μm long, amphidial fovea with 3.5–4 turns, lacking precloacal supplement	***D. nini***
–	Spicules longer than 100 μm, amphidial fovea less than three turns, precloacal supplement present	**23**
23	Body length shorter than 1750 μm, cephalic setae 5–7 μm long	***D. lutosa***
–	Body length longer than 2000 μm, cephalic setae shorter than 5 μm	**24**
24	Excretory pore anterior to nerve ring, buccal cavity 28–33 μm long	***D. jinmendaoica***
–	Excretory pore posterior to nerve ring, buccal cavity 18–21 μm long	***D. papilla***
25	Cuticle with two rows of coarse dots in males and three in females	**26**
–	Cuticle with more than two rows of coarse dots in males	**27**
26	Spicules distally acute, without hook	***D. poriferum***
–	Spicules distally with a subterminal hook	***D. timmi***
27	Cuticle with three rows of coarse dots in middle body region of males	**28**
–	Cuticle with four or five rows of coarse dots in middle body region of males	**31**
28	Tail with swollen tip and terminal setae	**29**
–	Tail without swollen tip and terminal setae	**30**
29	Body 4.8–5.6 mm long, spicules 220 μm	***D. janetae***
–	Body shorter than 2.5 mm, spicules 109 – 118 μm	***D. zhangi* sp. nov**.
30	Tail longer, 11–14 abd	***D. halongensis***
–	Tail shorter, 5–7 abd	***D. boucheri***
31	Spicules without subterminal protrusion	***D. magellanense***
–	Spicules with subterminal protrusion	***D. angelae***

## Conclusion

The present work describes two new species of the genus *Dorylaimopsis* from the Yellow Sea, China. As a result, 31 valid species within the genus have now been described. Accordingly, the number of species found in the Chinese sea area has increased from seven to nine. Based on the identification and analysis of *Dorylaimopsis* species, the main interspecific differences are the arrangement of lateral differentiation of the cuticle, as well as the structure and size of the spicules and gubernaculum. In addition, body size, the number of turns of the amphidial fovea, and the shape and length of the tail are also important criteria. *Dorylaimopsis* species exhibit a broad geographic distribution in marine sediments worldwide, from the intertidal zone to abyssal plains. *Dorylaimopsis* shows notable regional endemicity, with some species restricted to specific sea areas such as the South China Sea, East China Sea, or the Arctic Ocean. Most species are associated with organic-rich, fine sand or muddy substrates.

## Supplementary Material

XML Treatment for Dorylaimopsis

XML Treatment for Dorylaimopsis sinica

XML Treatment for Dorylaimopsis zhangi

## Additional information

### Artificial Intelligence (AI) use

The authors accept full responsibility for the content of the manuscript, including the disclosure of any use of AI.

No AI tools were used in the preparation of this manuscript.

### Author ORCIDs

M. Huang https://orcid.org/0000-0003-3343-1520

X. Li https://orcid.org/0009-0005-5036-865X

### Data availability

All of the data that support the findings of this study are available in the main text.
